# Modeling Development of a Diamagnetically Stabilized Magnetically Levitated Gravimeter

**DOI:** 10.3390/s24020350

**Published:** 2024-01-06

**Authors:** Kazi Rifat Bin Rafiq, Abigail Joseph, Naiya Yokochi, Peter James, Annette von Jouanne, Alex Yokochi

**Affiliations:** 1 Department of Mechanical Engineering, Baylor University, Waco, TX 76798, USA; kazirifatbin_rafiq1@baylor.edu (K.R.B.R.); abby_joseph1@baylor.edu (A.J.); naiya_yokochi1@baylor.edu (N.Y.); 2 Department of Geosciences, Baylor University, Waco, TX 76706, USA; p_james@baylor.edu; 3 Department of Electrical and Computer Engineering, Baylor University, Waco, TX 76706, USA; annette_vonjouanne@baylor.edu

**Keywords:** diamagnetic stabilized levitation, pyrolytic graphite, magnetic susceptibility, buoyancy, spring constant, gravimeter

## Abstract

The aim of this work is to create a new type of gravimeter that can function effectively in the challenging conditions of space, specifically on the surfaces of planets and moons. The proposed device, called a diamagnetically stabilized magnetically levitated gravimeter (DSMLG), uses magnetic forces to balance a test mass against the force of gravity, allowing for accurate measurements. A diamagnetically stabilized levitation structure comprises a floating magnet, diamagnetic material, and a lifting magnet. The floating magnet levitates between two diamagnetic plates without the need for external energy input due to the interaction between the magnetic forces of the floating magnet and the stabilizing force of the diamagnetic material. This structure allows for stable levitation of the floating magnet without requiring additional energy. The goal is to design a gravimeter that is lightweight, requires minimal power, can withstand extreme temperatures and shocks, and has a low data rate. The authors envision this gravimeter being used on various robotic spacecraft, such as landers and rovers, to study the interiors of rocky and icy celestial bodies. This paper reports on the results of a finite element model analysis of the DSMLG and the strength of the resulting diamagnetic spring. The findings contribute to the understanding of the levitation characteristics of diamagnetically stabilized structures and provide valuable insights for their practical applications, including in the development of the proposed DSMLG.

## 1. Introduction

Gravity is a valuable means of determining the distribution of mass inside a planet. Currently, gravity information for celestial bodies other than Earth is mainly obtained from orbiting satellites via Doppler ranging and satellite-to-satellite tracking. A gravimeter situated on the surface would provide measurement abilities that cannot be achieved from orbit.

Satellites can measure gravity on a global scale but not at finer length scales. The Gravity Recovery and Interior Laboratory (GRAIL) mission to the Moon [1] collected the highest-resolution gravity data to date but still had limitations in spatial resolution due to attenuation [2]. Thus, surface gravimetry will be necessary to determine gravity anomalies at scales less than several kilometers. Typically, spacecraft measure the gravity potential field outside a celestial body, using the Brillouin sphere as a reference point. The Brillouin sphere is the smallest sphere centered at the body’s barycenter that covers all its topography [3]. Any Doppler ranging or satellite-to-satellite tracking beyond this sphere cannot be used to confidently estimate gravitational potential at altitudes below it. The shape and gravitational field of a celestial body deforms in response to tidal forces [4], and these tidal responses produce signals that are measurable by a gravimeter [5]. While Love numbers have been estimated from orbit and from ground displacement measurements [6], a gravimeter would provide an independent coupled estimate of the *k* and *h* Love numbers.

It is difficult to measure gravity inside the Brillouin sphere from orbit due to external potential divergence [7,8,9], and surface gravity measurements have large uncertainties. Measuring absolute gravity would require calibration on Earth and precise measurements during launch. However, a relative gravimeter on a rover could still provide useful data by taking multiple measurements over longer distances.

As a result, the gravitational Love numbers of different bodies are not well-constrained for spherical harmonic degrees greater than 2 [10,11]. A gravimeter that remains stationary on the surface of a body could more accurately detect the changes in gravitational acceleration caused by tidal deformation. Having such a system deployed could also help make other measurements, like detecting lava tubes on the moon, estimating the local terrain of a planet, and generally help understand the planetary internal structure.

Lava tubes, void spaces resulting from volcanic activity, create a characteristic gravity deficit [12]. The width of these tubes can be inferred from the shape of the observed gravity anomaly, although this gets complicated with non-cylindrical tube shapes. Lava tubes are larger on bodies with weaker gravity, such as the Moon, where they could reach widths of a few kilometers [13]. The radiation shielding provided by the ceiling of a lava tube makes these structures a suitable base for human exploration and settlement [14]. While lava tubes on the Moon are difficult to identify from orbit, a gravimeter mounted on a rover would be an effective means of detecting them [15,16]. Surface-based gravimetry could plausibly detect lunar lava tubes by measuring relative gravity with an error of less than 20 mGal [17], where 1 Gal, sometimes called a galileo after Galileo Galilei, is a unit of acceleration commonly used in precision gravimetry and is defined as 1 cm per second squared (1 cm/s^2^) [18].

Gravity anomalies can be used to estimate the bulk density of a planetary body’s local terrain, which can be determined using the Nettleton–Parasnis method [19,20,21,22]. Measuring bulk density at different scales and locations can reveal vertical profiles and three-dimensional variations [23,24]. Bulk density can be used to investigate mineralogy, the presence of dense igneous bodies [25], the presence of ice [26], and porosity, which is dependent on impact history and regolith formation processes [27,28,29]. Sedimentary rock density on a planet like Mars can provide insights into deposition methods and depth of burial [30,31]. Gravity signals associated with many of these phenomena exceed 1 mGal.

Tidal deformation is a powerful tool for understanding a planetary body’s internal structure and can reveal information such as the size of a liquid metal core [6] or the presence of subsurface oceans [32,33,34]. It can also indicate tidal dissipation [35] and geophysical phenomena, such as volcanism [36], geysers [37], ocean [38], and energetic conditions that could be suitable for microbial life [39]. Tidal tomography [40], which maps the deep interior of a planetary body, is a promising technique that could reveal heterogeneities caused by magma ocean overturn and thermochemical convection [41,42,43,44]. Hemispheric dichotomies in surficial geology are a topic of interest in planetary science, and tidal deformation may offer insights into their origins. By measuring gravity at a single location, surface-based gravimetry can measure a tidal gravity perturbation (<200 μGal for a full tidal cycle). Ultra-high-precision measurements could potentially resolve deep mantle heterogeneities (<0.3 μGal for a full tidal cycle) [45] or detect earthquakes instantaneously (<1 μGal) [46]. 

The acceleration of gravity can be measured using various instruments, including the free-fall of a test mass in a vacuum and superconducting levitation of a test mass [47,48], but they are too massive for field geophysics or spacecraft missions. The most common type of gravimeter used in terrestrial geophysics is the spring-based gravimeter, which measures changes in gravitational acceleration in time and space [49]. However, these instruments have limitations for scientific investigations beyond Earth. An example of a commercially available instrument is the Scintrex CG-6 Autograv gravimeter, which uses a fused quartz spring [50]. 

A history of the development of gravimetry and applications to geodesy ranging from Harrison’s and Borda’s Pendulum systems in the 18th Century to Cavendish’s Free Fall Experiments in the 19th Century, to the development of Spring Gravimeters by researchers like Jeffreys and Harris, culminating in the more recent Superconducting Gravimeters (~1970s) like the GWR iGrav and the Scintrex systems can be found in [51,52,53,54]

Clearly, a variety of gravimeter designs have been built, and an even greater diversity of designs have been proposed. Global gravity fields can be recovered by orbiting spacecraft with a variety of detection techniques (e.g., GOCE, GRACE, and GRAIL), but these datasets are practically limited in their horizontal resolution by their altitude. For Earth, the finest resolution of orbitally measured static gravity fields is a few hundred kilometers [55]. Consequently, ground-based gravimeters are needed to map shorter-wavelength anomalies. Whereas “absolute” gravimeters directly measure the amplitude of gravity acceleration (e.g., through free fall), relative gravimeters like the DSMLG measure relative changes in acceleration; relative gravimetry is more practical for field deployment and is the focus of our paper.

In a spring-based instrument, changes in temperature can significantly affect the restoring force of the mechanical spring. To mitigate this, some modern gravimeters use fused quartz springs with low thermal expansion coefficients. However, temperature fluctuations can still lead to large changes in apparent gravity readings. 

To overcome the temperature sensitivity, these gravimeters are typically heated to maintain a constant temperature, causing them to require significant energy and power resources to accomplish this. For example, the Scintrex CG-6 gravimeter weighs 5.2 kg without an autonomous leveling system, with the weight due in part to the instrument’s thermal regulation components. These requirements would be even higher to maintain a constant temperature on the moon. 

Finally, during launch, separation, entry, descent, and landing, springs in gravimeters can experience an elastic change in length, known as “tares.” In addition, delicate springs can be damaged if the gravimeter is inverted while the test mass is unlocked. Locking and unlocking the test mass can also introduce tares in the spring [56]. The most common relative gravimeter design balances the force of gravity against known elastic stresses, including a zero-length spring or a vibrating string [49,57]. Micro-electromechanical systems (MEMS) similarly balance the strength of gravity against elasticity, and gravimeters based on these principles have made great strides in recent years [58].

All elasticity-based gravimeter sensors suffer from similar limitations, including temperature sensitivity, ambient noise, and instrument drift. Sensors based on electromagnetic forces could plausibly exhibit improved performance regarding these limitations and may, therefore, be desirable for some applications. A gravimeter that uses electrostatic forces has been proposed [59], but this design still incorporates an elastic spring. Superconducting gravimeters do not rely on elasticity, but they are bulky and impractical for mobile deployment. Compared to existing alternatives, a gravimeter that incorporates diamagnetic levitation would potentially have the benefit of improved stability, reduced noise, improved sensitivity, and operation at room temperature [60].

**Figure 1:**
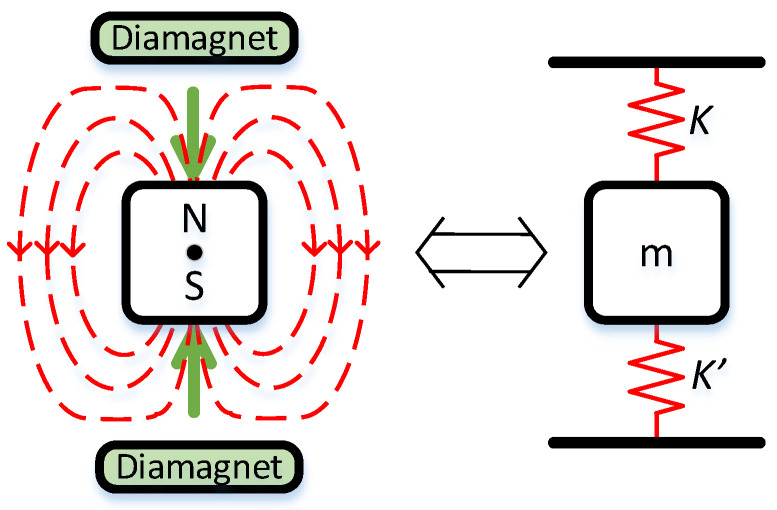
The diamagnetic plates expel the magnetic field (dashed red lines) from its bulk, deforming the magnetic field and creating a repulsive magnetic force (green arrows) between the magnet and the diamagnetic plates (**left**). This is equivalent to the presence of magnetic springs with force constants k and k’, whose force constant is dependent on the spacing between the face of the magnet and the diamagnetic plates (**right**).

To address these issues, we have started the theoretical development of a diamagnetically stabilized magnetically levitated gravimeter (DSMLG), which uses a magnetic spring, shown diagrammatically in Figure 1, resulting from the interaction of the magnetic fields of permanent magnets and diamagnetic materials. This system should be less sensitive to drift in response to stresses than a mechanical spring [61], have much lower temperature sensitivity, and consequently much lower energy and power requirements to take similarly reliable gravity measurements, which in turn simplify deployment and prolong operational lifetime. While the practical design of such an instrument is beyond the scope of this paper, the sensor casing could inductively dampen the motion of the test mass through eddy current braking, which would mitigate ambient noise experienced by elasticity-based sensors.

The DSMLG is based on the stable levitation properties of a structure developed [62] through the investigation of the levitation properties of the floating magnet within a diamagnetically stabilized levitation system. The floating magnet in the diamagnetically stabilized levitation structure exhibits three distinct levitation states—symmetric monostable levitation, bistable levitation, and asymmetric monostable levitation—with our main focus on the symmetric monostable levitation mode [63]. This study presents the results of simulations to explore the levitation characteristics of the structure, and in particular, the effect of adjusting the gap between diamagnets and magnets on the resulting magnetic spring Hooke’s Law constant.

## 2. Methodology

### 2.1. Proposed Gravimeter Device 

The diamagnetically stabilized magnetically levitated gravimeter (DSMLG) is a levitation gravimeter that utilizes the attraction between two permanent magnets to oppose the average gravitational force experienced by the test mass at the deployment location. The test mass, a permanent magnet, is levitated by the magnetic field of a fixed magnet, and the only significant force acting on the test mass will be diamagnetic repulsion from diamagnetic plates.

**Figure 2:**
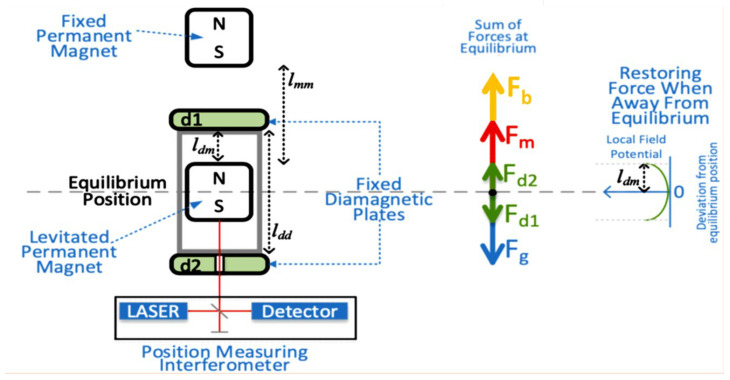
A diagram of the developed gravimeter device (**left**) and of the balance of forces acting on the test mass (**right**).

A diagram of the device under examination, shown in Figure 2, depicts the levitated permanent magnet (i.e., the test mass), the fixed permanent magnet that opposes the gravitational force on the test mass, and the two diamagnetic plates that repel the levitated permanent magnet. When at equilibrium, the sum of the forces acting on the test mass, viz. the gravitational force ( $F_{g}$ ), magnetic force ( $F_{m}$ ), and the two diamagnetic repelling forces ( $F_{d1}$ and $F_{d2}$ ), sum to zero. When the test mass is displaced from the equilibrium point, it experiences a restoring force equal to the sum of the two diamagnetic repelling forces. The test mass cannot move more than the distance between the diamagnetic plate and the levitated magnet ( $l_{dm}$ ) due to physical limitations. The separation between the two diamagnetic plates ( $l_{dd}$ ) controls the depth of the potential well in the diamagnetic repulsion field, allowing for a softer or harder “diamagnetic spring” by adjusting this distance.

We also plan to experimentally validate the model, with results to be shared in an upcoming paper. To verify the results, we will change the summation of forces at the test mass. To accomplish this, rather than attempting to modify the force of gravity (which is generally not possible), we can change the density of the surrounding medium, thereby changing buoyancy forces on the test mass ( $F_{b}$ ). This was deemed a better approach to examine the reliability of the model rather than attempting to modify the strength of the fixed permanent magnet, whether by adding permanent magnet fragments or by adding an electromagnet to the system.

Note that in order to enable measuring the position of the levitated permanent magnet using a white light interferometer, we also need to introduce a small bore in the lower fixed diamagnetic plate, the effect of which also must be studied.

### 2.2. Basic Principles of Diamagnetically Stabilized Magnetic Levitation

The history of magnetism can be traced back to ancient Greece, where the philosopher Thales of Miletus observed that lodestone (a naturally occurring magnet) could attract iron [64]. In the 16th century, William Gilbert, an English physician, conducted extensive experiments and published a treatise on magnetism, which laid the foundation for the modern understanding of magnets. Diamagnetism, on the other hand, was first observed and studied by Michael Faraday in the early 19th century, who noticed that certain materials weakly repelled a magnetic field [65]. This discovery paved the way for the understanding of the fundamental properties of materials and their interaction with magnetic fields.

The magnetic energy of an object of volume *V* and magnetic susceptibility *χ* in a field of magnetic flux density $\vec{B}$ is given by:

(1) $$E_{mag}=-\frac{1}{2\mu }\;\chi VB^{2}$$

and since $\vec{F}=\vec{\nabla }E$ , the magnetic force (in N) experienced by a magnetic system is:

(2) $${\vec{F}}_{mag}=\frac{\chi }{\mu }V\;\left(\vec{B}\cdot \;\vec{V}\right)\;\vec{B}$$

and depends on the magnetic susceptibility of the material, *χ* (nondimensional), its volume, *V* ( $m^{3}$ ), the magnetic flux density of the applied field, $\vec{B}$ (T), the gradient of the magnetic field, $\vec{B}\cdot \;\vec{V}\;$ (T/m), and the permeability of free space, $\mu_{0}=4\pi \times 10^{-7}$ H/m.

If an object is either ferromagnetic or paramagnetic (*χ* > 0), it will show a positive result with a positive value of magnetic force ( ${\vec{F}}_{mag}$ ), indicating that it is attracted to the magnetic field. On the other hand, if the material is diamagnetic (*χ* < 0), it will display a negative result with a negative magnetic force ( ${\vec{F}}_{mag}$ ), indicating that it is being repelled by the magnetic field. Essentially, materials that have a greater magnetic susceptibility than their surroundings are pulled toward high magnetic field areas, and conversely, materials with a magnetic susceptibility smaller than their surroundings are expelled from high magnetic field areas, which is a phenomenon that is not often observed directly.

It has been observed that magnetic objects can be trapped in stable locations, but only in areas where there is a maximum magnetic field. This means that materials with greater magnetic susceptibility than their surroundings can only be stably trapped at the source of the magnetic field. However, magnetic field minima can be created outside of a magnetic field source, which allows for the levitation and confinement of diamagnetic materials like biological materials. In contrast, ferromagnetic materials can be trapped between two diamagnetic plates at the minimum energy location created by the magnetic field. 

The proposed device relies on trapping a strong permanent magnet in the energy minimum between two diamagnetic plates (the location where $E_{mag}$ is a minimum according to Equation (1)), where the restoring forces are determined by the magnetic force as described by the equation. This means that any deviation of the object from the minimum energy location will result in a magnetic force ( ${\vec{F}}_{mag}$ ) that acts to restore it to that location.

### 2.3. Mathematical Foundations

**Figure 3:**
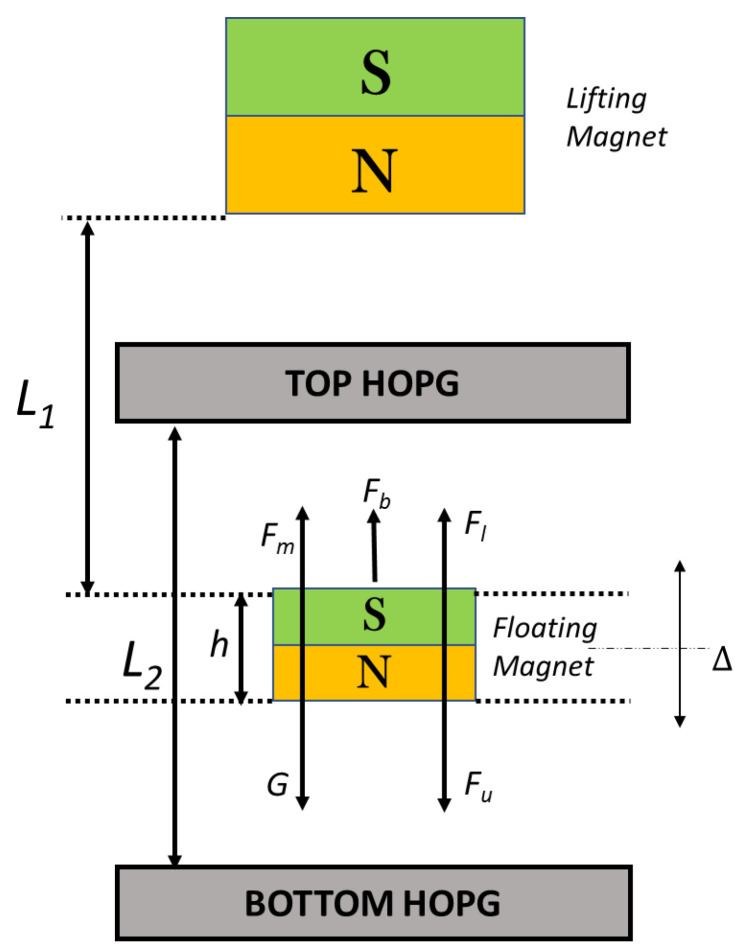
A schematic of the diamagnetically stabilized magnetically levitated gravimeter. The delta (Δ) refers to the displacement from the equilibrium position indicated by the thin dashed line.

The resultant force $F_{r}$ for the system shown in Figure 3 is given as

(3) $$F_{r}=F_{m}+F_{l}-F_{u}-G$$

where $F_{m}$ is the force exerted on the floating magnet by the lifting magnet, $F_{l}$ and $F_{u}$ are the lower and upper opposite repulsive forces exerted on the floating magnet by two highly oriented pyrolytic graphite (HOPG) sheets, and G is the gravitational force on the floating magnet.

A study is proposed to investigate the effect of buoyancy on the resultant force in the case that the chamber pressure is above vacuum such that the new resultant force $F_{r}^{*}$ includes the buoyancy force, i.e.,

(4) $$F_{r}^{*}=F_{m}+F_{l}-F_{u}-G^{*}$$

(5) $$G^{*}=G-F_{B}$$

$F_{B}$ is the buoyancy force.

(6) $$G=mg=\rho Vg,\;F_{B}=\rho^{*}Vg,\;G^{*}=\left(\rho -\rho^{*}\right)Vg$$

$\rho^{*}$ is the density of the medium,

(7) $$G^{*}=\rho Vg^{*}$$

where $g^{*}$ is the effective local gravitational acceleration

(8) $$g^{*}=\left(\frac{\rho -\rho^{*}}{\rho }\right)g$$

The radial, *B_r_*, and axial, *B_z_*, magnetic field components described in an axisymmetric cylindrical coordinate system, therefore, defined only by a radial, *r*, and height, *z*, coordinate for a magnet with magnetic dipole moment, $M_{d}$ , immersed in a medium with the magnetic permeability of vacuum, $\mu_{0}$ , is given analytically by

(9) $$B_{r}^{\;}\left(r,z\right)=\frac{\mu_{0}M_{d}}{4\pi }\left[3\frac{rz}{a^{5}}\right],\;B_{z}^{\;}\left(r,z\right)=\frac{\mu_{0}M_{d}}{4\pi }\frac{1}{a^{3}}\left[3\frac{z^{2}}{a^{2}}-1\right],\;\mathrm{and}\;a\left(r,z\right)=\sqrt{r^{2}+z^{2}}$$

We compute the minimum $L_{1}$ from the balance of forces, i.e.,

(10) $${\left.B^{\prime }\right|}_{r=0,z}=\frac{mg}{M_{d}}\;\overset{find\;z}{\to }\;L_{1}\;$$

where,

(11) $$B^{\prime }=\frac{\partial B_{z}}{\partial z}=3\frac{\mu_{0}M_{d}}{4\pi }\frac{1}{a^{7}}\left(3r^{2}z-2z^{3}\right)$$

(12) $$L_{1}^{*}=\sqrt[{4}]{\frac{3\mu_{0}}{2\pi }\frac{M_{d}^{2}}{mg}}$$

For vertical and horizontal levitation stability,

(13) $$L_{2}^{\;}<{\left\{\frac{12\mu_{0}M_{d}\left|\chi \right|}{\pi B^{\prime \prime }}\right\}}^{\frac{1}{5}}<{\left\{\frac{24\mu_{0}B_{0}{M_{d}}^{3}\left|\chi \right|}{\pi {\left(mg\right)}^{2}}\right\}}^{\frac{1}{5}}$$

where $M_{d}$ and m are the magnetic dipole moment and mass of the floating magnet.

(14) $$M_{dd}=MV,\;M_{d}=\left|M\right|V$$

${\vec{M}}_{\;}$ is the magnetization of the magnet, *V* is the volume,

(15) $$\left|M\right|=Br/\mu_{0},\;V=\frac{1}{4}\pi d^{2}h$$

(16) $$B_{0}=\frac{\mu_{0}}{\pi }\left|M\right|=\frac{Br}{\pi }$$

(17) $$B^{\prime \prime }=\frac{\partial^{2}B_{z}}{\partial z^{2}}=3\frac{\mu_{0}M_{d}}{4\pi }\frac{1}{a^{9}}\left(3r^{4}-24r^{2}z^{2}+8z^{4}\right)$$

(18) $$\mu_{0}=4\pi \times 10^{-7}{\mathrm{NA}}^{-2}$$

The relative susceptibility is $\mu_{r}=1+\chi$ .

Based on a crude estimate of the force on the magnet using the image method it can be obtained from

(19) $$F_{d}=3\frac{\mu_{0}\chi_{z}}{4\pi }{M_{d}}^{2}\frac{1}{a^{4}}$$

To obtain the tangent stiffness at the equilibrium point, a hyperbolic sine function fit was used to approximate each F−Δ curve, where Δ represents the displacement of the levitated magnet from the equilibrium point, as shown in Figure 3. The stiffness is obtained by computing the first-order derivative of the hyperbolic function at the zero-crossing ( Δ=0 ). We define the hyperbolic function relating the force, F, to the displacement, Δ, using parameters *a*_0_, *a*_1_, and *a*_2_, and the resulting value of the gradient of the force vs. displacement, as shown in Equation (20)

(20) $$F_{i}=a_{0}+a_{1}\sinh\left(a_{2}\Delta_{i}^{\;}+a_{3}\right)\;\Rightarrow \;{\left.\frac{\partial F}{\partial \Delta }\right|}_{i}=F_{i}^{’}=a_{1}a_{2}\cosh\left(a_{2}\Delta_{i}^{\;}+a_{3}\right)\;$$

(21) $$K={\left.F_{i}^{’}\right|}_{\Delta =0}=a_{1}a_{2}\cosh\left(a_{3}\right)$$

The universal gravitational constant $\tilde{G}$ can likewise be obtained from the force–displacement relationship based on Newton’s law of universal gravitation, given as

(22) $$F\left(\Delta \right)=\tilde{G}\frac{m\cdot \tilde{m}}{{\left(L_{1}^{*}+\Delta \right)}^{2}}=m\cdot g,\;g=\frac{F\left(\Delta \right)}{m}=\frac{\tilde{G}\tilde{m}}{{\left(L_{1}^{*}+\Delta \right)}^{2}}$$

where $\tilde{m}$ is the mass of the lifting magnet, and $\tilde{G}$ is the gravitational constant.

### 2.4. Model Implementation

The study employed finite element analysis (FEA) simulation using COMSOL Multiphysics 6.0 to determine the resultant force. The geometric model used was an axisymmetric model for 2D analysis. The simulation used the structure parameters listed in Table 1 and calculated the magnetic force between magnets and the diamagnetic force between the magnet and the diamagnet to obtain the movement space. The impact of structural parameters on the movement space of the floating magnet was analyzed, and the experimental results confirmed the accuracy of the simulation.

**Figure 4:**
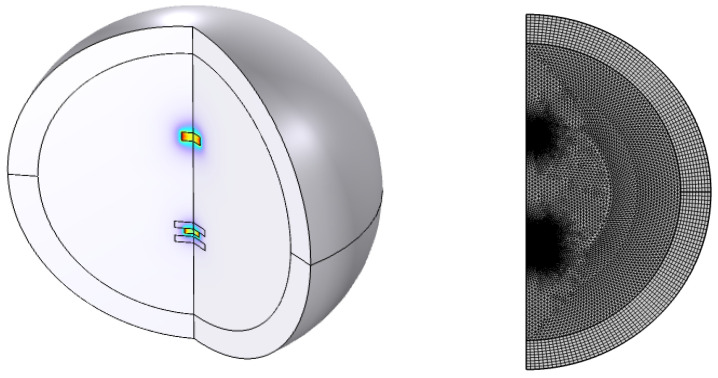
Finite element analysis (FEA) model of the diamagnetically stabilized levitation gravimeter (DSMLG). The top fixed levitating magnet, the fixed diamagnetic plates, and the movable levitated magnet are embedded in a general non-magnetic medium of density $\rho^{*}$ . The central, linear region, is surrounded by an “infinity shell” to minimize termination errors. The relative magnitude of the magnetic flux density is indicated by the blue color in the figure on the left side, and the finer meshing used in the space close to the magnetic materials (magnets and diamagnetic plates) is shown on the right.

The magnetic and diamagnetic forces were calculated using a stationary study in COMSOL Multiphysics. The free-meshing algorithm using triangular elements was applied to all domains except the infinite domain region, which was mapped with a mesh of 10 elements. The maximum element size of the magnets and pyrolytic graphite sheets was set at 1.5 mm, and the meshing scale of the air domain was set to “Extremely fine” with a 2.45 mm element size. The simulation model had approximately 11,510 triangular elements in the two meshed magnets, and the elements of air surrounding the two magnets were refined to match those of the magnets. The solution time of the model on an Intel(R) Xeon(R) Gold 6136 CPU 3 GHz and 256 GB RAM computer was 53 s to complete the simulation for each $L_{2}$ distance. The simulation model is depicted in Figure 4.

## 3. Results and Discussion

### 3.1. Initial Sensitivity Analysis

**Figure 5:**
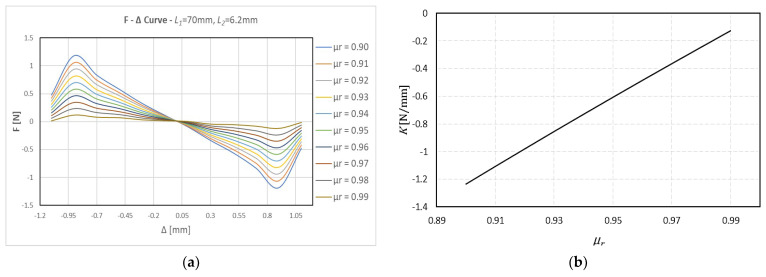
(**a**) Resultant force vs. displacement curves for different relative permeability ( $L_{1}$ = 70 mm and $L_{2}$ = 6.2 mm). (**b**) Spring constant with respect to the corresponding relative permeability $\mu_{r}$ .

For calibration purposes, an initial sensitivity analysis was carried out for various relative permeability values of the HOPG ( $\mu_{r}^{\;}$ ) ranging from 0.90 to 0.99 in step size of 0.1 using a magnetic spacing ( $L_{1}$ ) of 70 mm and a diamagnetic spacing ( $L_{2}$ ) of 6.2 mm. The results of the sensitivity analysis are shown in Figure 5a, showing that higher relative permeability leads to a flatter resultant force–displacement ( F−Δ ) curve with smaller diamagnetic end repulsive forces and vice versa. The spring stiffnesses of each ( F−Δ ) curve (excluding the end repulsive forces) are obtained from linear regression analysis, and the results presented in Figure 5b show a linear dependence of the resulting spring stiffness on the relative permeability. For prototyping purposes, we require a diamagnet with high relative permeability close to unity since we expect to operate with magnetic forces in the order of 0.1 N.

**Figure 6:**
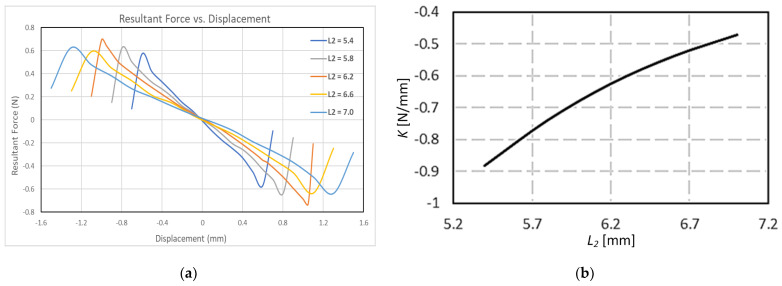
(**a**) Resultant force with respect to various displacements for different gaps $L_{2}$ ( $L_{1}$ = 70 mm and μ=0.95 ). (**b**) Spring constant with respect to the corresponding $L_{2}$ displacement.

A further sensitivity analysis was conducted to study the effect of varying the diamagnetic spacing ( $L_{2}$ ) on the characteristic ( F−Δ ) curve. In this study, we assume a relative permeability $\mu_{r}$ of 0.95, and a magnetic spacing $L_{1}$ of 70 mm. A range of $L_{2}$ distances from 5.4 mm to 7.0 mm with a step size of 0.4 mm was used for this parametric study. The results of the analysis (cf. Figure 6a,b) show that reducing the diamagnetic spacing ( $L_{2}$ ) increases the spring stiffness and the accompanying diamagnetic end repulsive forces.

**Figure 7:**
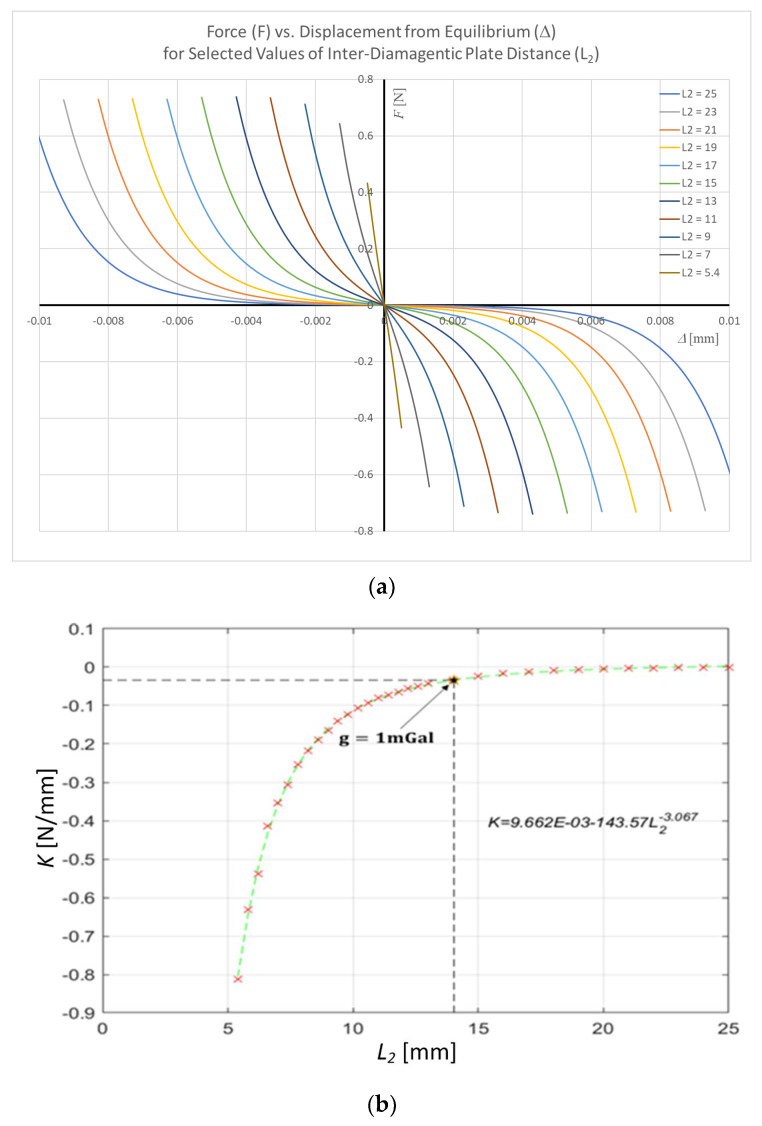
(**a**) Resultant force with respect to various displacements for wide range of $L_{2}$ distances from 5.4 mm to 25 mm ( $L_{1}$ = 70 mm and μ=0.95 ). (**b**) Spring constant gradient with respect to the spacing between the two diamagnetic plates $L_{2}$ (for the same $L_{1}$ = 70 mm and μ=0.95 case). Red crosses mark points at which the model was run, and the dashed green line shows the power curve for the equation shown in the figure. The black dashed lines show the hypothetical case where a change in gravitational acceleration of 1mGal results in a displacement of 1 micron for a test mass of 3.4 g.

As shown in Figure 6, the variation of the spring stiffness K, with the diamagnetic spacing $L_{2}$ is not linear. To obtain a better understanding of the general relationship between the spring stiffness K and the $L_{2}$ distance between the two diamagnetic plates, an additional parametric study involving a wide range of $L_{2}$ distances (5.4 mm to 25 mm) was carried out. The study implemented a systematic approach, employing a step size of 0.4 mm within the range of 5.4 mm to 13 mm. Subsequently, a larger step size of 1 mm was adopted from 13 mm to 25 mm. Figure 7a shows that for higher $L_{2}$ distances, the nature of characteristic ( F−Δ ) curves are non-linear. The spring stiffness for each ( F−Δ ) curve is determined by obtaining the tangent stiffnesses at the neutral axis of the hyperbolic sine function regression fit approximations of actual ( F−Δ ) curves (excluding the end repulsive forces) based on the methodology presented in the preceding section. The resulting profile of the calculated spring stiffness for each diamagnetic spacing $L_{2}$ is shown in Figure 7b. A power-law fit to the calculated spring-stiffness K, and diamagnetic spacing $L_{2}$ , yields Equation (23) below.

(23) $$K=0.0097-143.57{L_{2}}^{-3.067}$$

We observe from the trend that as $L_{2}$ approaches 0, the spring stiffness, K, approaches infinity, and vice versa, i.e.,

(24) $${\left.K\right|}_{L_{2}\to 0}=-\infty ,\;{\left.K\right|}_{L_{2}\to \infty }=0$$

The results in Figure 7b show that we can tune the magnetic spring constant to an arbitrarily low value with which to construct a high-precision gravimeter by changing the diamagnetic spacing ( $L_{2}$ ). This also enables us to carry out some initial gravimeter design activity. Say we desire to measure a gravitational change of 1 mGal that results in a displacement of the test mass by 1 micron, corresponding to a spring stiffness of $1\;\mathrm{mGal}\times m_{t}=K\times 1\mu m\;\Leftrightarrow \;10\left[\frac{{\mathrm{mm}}^{2}}{s}\right]\times m_{t}=K\times 10^{-3}\;\left[\mathrm{mm}\right]$ where $m_{t}$ is the mass of the floating magnet, which, in our case, is $m=3.4\times 10^{-3}\;\mathrm{kg}$ . From the derived stiffness–diamagnetic spacing relationship (cf. Equation (23)), we require a gravimeter with a diamagnetic spacing of 14.03 mm.

### 3.2. Multidimensional Force–Displacement Parametric Study

**Figure 8:**
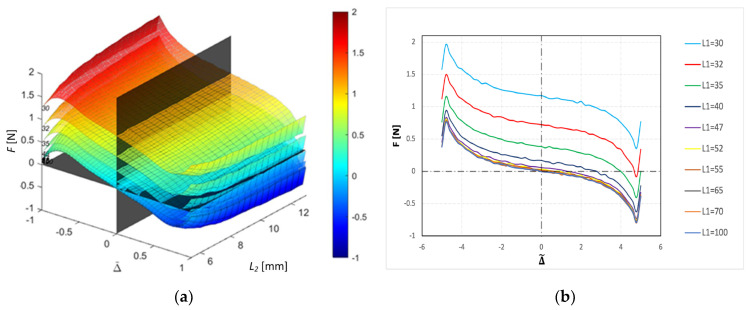
(**a**) A 3D surface plot of resultant force vs. displacement (*x*-axis) for different $L_{2}$ (*y*-axis) and $L_{1}$ (*z*-axis) distances. (**b**) Plot of resultant force vs. displacement for $L_{2}$ = 14 mm (corresponding to 1 mGal). $\tilde{\Delta }$ represents the normalized displacement and is given by $\tilde{\Delta }=\Delta /\Delta_{L_{2}}$ , where ΔL2=12L2−h .

**Figure 9:**
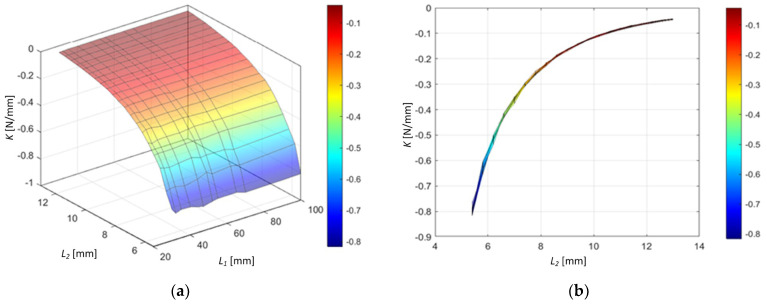
(**a**) The 3D spring stiffness response surface with $L_{1}$ and $L_{2}$ parameter dependence; (**b**) sideview of the 3D stiffness response surface.

Here, we study the effect of a multidimensional parametric variation of the magnetic spacing $L_{1}$ and diamagnetic spacing $L_{2}$ on the resultant force–displacement response to obtain the general characteristics of the stiffness behavior for a wide range of levitation configurations. We assume a relative permeability of 0.95 for the HOPG diamagnets in this study. Figure 8a shows the results of different force–displacement–diamagnetic spacing ( $F-\Delta -L_{2}$ ) response surfaces for various magnetic spacings $L_{1}$ ranging from 30 mm to 100 mm. A sideview (cf. Figure 8b) of the 3D surfaces gives a better representation of the ( F−Δ ) response variation with the $L_{1}$ distance. The results show that we attain stable equilibrium with a magnetic spacing of $L_{1}^{*}$ of 47 mm, beyond which stability conditions of magnetic levitation remain unaffected. From Equation (12) above, the minimum $L_{1}$ distance for stable levitation was obtained as $L_{1}^{*}=47.3$ mm, which corroborates our findings. As $L_{1}$ drops below $L_{1}^{*}$ , the ( F−Δ ) curves drift farther away from the equilibrium position. In Figure 9, we show different 3D slices of the force–displacement (F-Δ) response surfaces along the $L_{2}$ -axis to show that the optimum magnetic spacing for stable levitation $L_{1}^{*}$ is unaffected by the $L_{2}$ distance.

We obtain a 3D spring stiffness response surface (cf. Figure 9a) with $L_{1}$ and $L_{2}$ parameter dependence using the same tangent stiffness algorithm of the hyperbolic sine function regression fit approximations for the different ( F−Δ ) responses of the various $L_{1}$ and $L_{2}$ combinations. A sideview (cf. Figure 9b) of the 3D stiffness response surface shows that the spring constant is nearly independent of the magnetic spacing $L_{1}$ but varies non-linearly with distance $L_{2}$ between the two HOPG diamagnets. As such, the spring stiffness–diamagnetic spacing correlation equation derived previously (cf. Equation (23)) is sufficient in describing the 3D response behavior without considering the $L_{1}$ parameter in its definition.

### 3.3. Effect of Diamagnetic Bore on the Characteristic ( F−Δ ) Curve

To enable measurement of the equilibrium position of the floating magnet, provisions must be made for the laser beam of an interferometer to pass through. This beam is approximately 0.5 mm in diameter. This is achieved by creating a small, centered bore in the base HOPG diamagnet. The bore is expected to slightly alter the characteristic ( F−Δ ) curve depending on the bore size. The sensitivity of the forces to the size of the bore is modeled to ensure a practical instrument can still be developed and determine an appropriate bore radius that would allow complete passage of the beam with slight tolerance for disturbance without significantly altering the characteristic ( F−Δ ) curve. 

**Figure 10:**
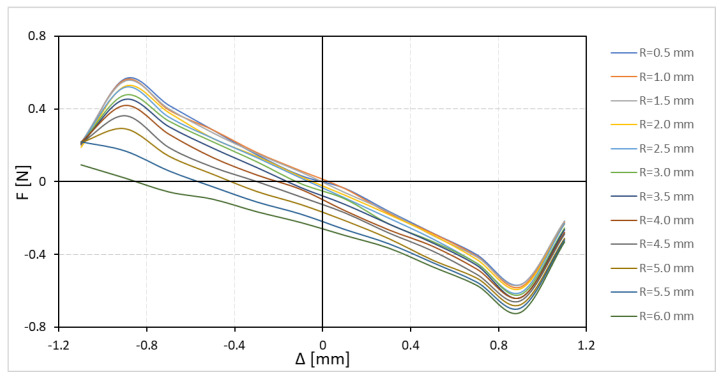
Force–displacement ( F−Δ ) characteristic profiles for different bottom HOPG diamagnetic bore radius R ( $L_{1}=70\;\mathrm{mm},\;\;L_{2}=6.2\;\mathrm{mm},\;\;\mu_{r}=0.95)$ .

In this study, a relative permeability $\mu_{r}$ of 0.95 was used for the HOPG diamagnets with a diamagnetic spacing $L_{2}$ of 6.2 mm and a magnetic spacing $L_{1}$ of 70 mm. From the result of the sensitivity analysis (cf. Figure 10), we see that with bore radius R , up to 2.0 mm, the characteristic ( F−Δ ) curve is not significantly affected. A further increase in the bore size results in an asymmetric placement of the floating magnet to attain a stable equilibrium. 

## 4. Conclusions

This paper presented a theoretical and computational investigation of the levitation characteristic of a diamagnetically stabilized levitation structure. Decreasing the spacing between the diamagnetic plates (*L*_2_ in the text) increases the spring constant and repulsive force, and conversely, increasing it will decrease the spring constant and can enable deploying a gravimeter with a spring constant as weak as necessary. 

Adding a bore in the lower diamagnetic sheet to enable the passage of the interferometer beam was also examined, and it was found that for a bore of radius up to 2.0 mm, little change in the magnet force constant was observed. However, an asymmetrical placement of the floating magnet (i.e., the distance between the levitated permanent magnet to the bottom diamagnetic plate is different than the distance to the top diamagnetic plate) is necessary for stable equilibrium when the diameter of the bore increases beyond one-third of the floating magnet’s diameter. These findings contribute to the understanding of the levitation characteristics of diamagnetically stabilized structures and provide valuable insights for their practical applications, such as the development of the proposed diamagnetically stabilized magnetically levitated gravimeter. 

## Figures and Tables

**Table 1:** Structure parameters of the diamagnetically stabilized magnetically levitated gravimeter.

Parameter	Lifting Magnet	Floating Magnet	Diamagnetic Sheet
Materials	NdFeB-52	NdFeB-52	HOPG
Size	Φ15×6.35 $\left[\mathrm{mm}\right]$	Φ12×4 $\left[\mathrm{mm}\right]$	Φ25×5 $\left[\mathrm{mm}\right]$
Residual Flux Density (Br)	1.45 $\left[T\right]$	1.45 $\left[T\right]$	-
Recoil permeability	1.05	1.05	-
Electrical conductivity	1/1.4 $\left[\mu \mathrm{ohm}\cdot m\right]$	1/1.4 $\left[\mu \mathrm{ohm}\cdot m\right]$	$3\times 10^{3}$ $\left[S/m\right]$
Desity		$7.5\times 10^{3}$ $\left[\mathrm{kg}/m^{3}\right]$	-
Relative permeability	-	-	0.95
Relative permittivity	-	-	1

## Data Availability

Data necessary to reconstruct the model used are contained within the article.
